# Effects of acute and chronic exercise on working memory in healthy adults. An experimental investigation

**DOI:** 10.3389/fpsyg.2025.1608721

**Published:** 2025-06-06

**Authors:** Eva Borrega-Alonso, F. Javier Otamendi

**Affiliations:** Department of Applied Economics I and History and Economic Institutions, School of Economics and Business, Universidad Rey Juan Carlos, Madrid, Spain

**Keywords:** exercise, working memory, chronic exercise, acute exercise, cognition, high-intensity functional training

## Abstract

**Introduction:**

Working memory is critical for optimal development of academic and occupational functions, and its deterioration is associated with mental health issues. Exercise-based interventions have emerged as a prominent strategy to enhance cognitive functions and improve mental health. The objective of this study is to analyze the effects of both acute and chronic physical exercise on working memory in the context of cognitive and experimental psychology.

**Methods:**

Participants (73 adults aged between 18 and 65 years) performed appraisal tests on working memory based on image recovery (Test 1) and decision-making on verbal mathematical operations (Test 2), with the experimental group performing one-hour intense functional training (acute exercise) immediately before. The World Health Organization (WHO) classification based on weekly chronic exercise was also used for categorization of participants.

**Results:**

The results show that both types of physical activity positively affect cognition. Chronic exercise favored all aspects of working memory (verbal mathematical operations and decision making; *p*-value = 0.014; pη^2^ = 0.081//image recovery; *p*-value = 0.033; pη^2^ = 0.062), while acute exercise only favored those related to image recovery (*p*-value = 0.007; pη^2^ = 0.099).

**Conclusion:**

To enhance working memory, it is recommended that both companies and educational centers promote both types of physical exercise. For future research, in addition to increasing the sample size, it would be beneficial to vary the length of training as well as the difficulty of the assessment tests.

## 1 Introduction

Any form of physical activity can activate the brain function, thereby facilitating the development of mental health in general and of numerous cognitive domains in particular. This, in turn, represents a crucial factor in growth (Serra et al., 2021; Mandolesi et al., 2018). More specifically, several studies have demonstrated that physical activity confers benefits on cognition (De Asteasu et al., 2017; Chang et al., 2012; Costa et al., 2019; Guiney and Machado, 2012; Hillman et al., 2008; Kamijo et al., 2009; Kao et al., 2019; Li et al., 2017; McMorris et al., 2011; Soga et al., 2018; Wilke et al., 2019). Cognition is defined as “all forms of knowledge and awareness, such as perceiving, conceiving, remembering, reasoning, judging, imagining, and problem solving” (APA Dictionary of Psychology, 2025).

The benefits of physical activity are especially evident in cognitive flexibility and working memory (Hsieh et al., 2020; Rathore and Lom, 2017), which is a main component of cognition and decision making. De Greeff et al. (2018) define working memory as the ability to actively store and manipulate task-relevant information in a short period of time, which is a central component of the executive function (Baddeley, 2010; Diamond, 2013).

Physical activity is usually related in this context to that performed regularly. We use the term *chronic exercise* in this research, taken from Gilson et al. (2023). Similar terms are regular exercise (Wu et al., 2024) or habitual exercise (Yuan et al., 2023). As such, the World Health Organization (WHO) recommends that adults between the ages of 18 and 64 engage in moderate aerobic physical test for at least 2.5 to 5 h per week or intense aerobic physical test for at least 1.25 to 2.5 h per week [World Health Organization (WHO), 2024]. We use these thresholds as benchmarks to categorize individuals.

However, there is another type of exercise, the one that might be performed immediately before an action that requires the use of the working memory. We use the term *acute exercise* in this research following Gilson et al. (2023). Acute exercise should also improve mental health and the cognitive tasks. Indeed, moderate-intensity continuous exercise training (MICE) and high-intensity interval exercise training (HIIT), both of which are somewhat similar to functional fitness training, improve working memory immediately after performing them (Mou et al., 2022; Wilke, 2020).

A few studies have investigated the association between high-intensity interval training (HIIT) and cognitive function. Yuan et al. (2023) found that greater amounts and higher frequencies of vigorous-intensity exercise were highly correlated with smaller recall errors in a working memory precision task. In a separate study, Connelly et al. (2017) reported that 12 weeks of HIIT and continuous training, including three 35-min sessions per week, led to improvements in memory. Concurrently, Eather et al. (2019) reported that three weekly sessions of 8–12 min of HIIT for eight weeks significantly enhanced cognitive flexibility. In a similar vein, Kovacevic et al. (2019) reported that a 12-week HIIT program comprising three 28-min sessions per week led to significant enhancements in high-interference memory. Additionally, Zhang et al. (2021) observed significant improvements in global cognitive function following a six-week intervention with two 60-min group HIIT sessions per week. These findings highlights the potential of HIIT as a promising strategy for enhancing various domains of cognitive performance.

A 12-min session of HIIT has been shown to enhance cognitive flexibility, inhibition, and reaction time when compared to a control group that was seated and resting (Solianik et al., 2021; Nasrollahi et al., 2022). Quintero et al. (2018) also demonstrated that a 32-min session of HIIT led to substantial enhancements in inhibition and attentional capacity. An additional study on the subject corroborated the findings, indicating that a 20-min session of HIIT enhanced information processing speed and inhibition in comparison to the resting group (Xie et al., 2020).

The objective of this study was to analyze the effect of both types of physical activity, chronic and acute exercise, on working memory. There is not a large body of scientific literature on the effects of both acute and chronic physical exercise. In their meta-analysis, Gilson et al. (2023), after analyzing 36 studies, including 22 on acute exercise and 14 on chronic exercise, concludes that while both types of exercise may have a positive impact on cognitive function, the evidence is more consistent for acute effects, particularly in tasks requiring attention and processing speed. However, studies on chronic exercise suggest potential long-term benefits, albeit with greater variability in results, highlighting the need for further research with standardized methodologies and larger sample sizes.

More specifically, the two main hypotheses are:

- [H1] Chronic exercise enhances working memory.
- [H2] Acute exercise enhances working memory.

To test the hypotheses, an experimental approach was followed. We measured working memory on individuals based on two appraisal tests, one related to visualization and recovery of images, and the other on mathematical processing and decision making, each one designed to address different components of working memory.

With respect to the quantification of *chronic exercise*, all of the participants had to declare via questionnaire how often they carry physical activities, so they were liable to be categorized according to the WHO classification.

To study the impact of *acute exercise*, a group of participants performed a novel training just before the appraisal tests while the rest of individuals had to rest at least in the previous two hours before taking the tests. Specifically, the type of acute exercise was functional fitness training, which is similar to Crossfit (registered trademark) and HIIT. High-intensity functional training (HIFT) has been demonstrated to improve cognitive performance, specifically working memory, with this benefit being slightly superior to aerobic walking exercise (Wilke, 2020). In this research, the length of the exercise is one-hour, longer than any other reported research in the related meta-analysis (between 6 and 40 min) (Mou et al., 2022; Wilke, 2020; Solianik et al., 2021; Nasrollahi et al., 2022; Xie et al., 2020). A 1-h session was chosen because the classes offered in gyms for this activity are usually of this duration.

## 2 Methods

### 2.1 Quantification of working memory

Baddeley (2012) proposed that working memory is composed of four components: the (1) phonological loop, which stores verbal or auditory information in a temporal manner; (2) the visuospatial sketchpad, which stores visual and spatial information in a temporal manner; (3) the episodic buffer, which integrates information from different sources and domains in a temporal and spatial manner; and (4) the central executive, which monitors and coordinates the activities of the phonological and visuospatial components, operating with such information.

For this research, two tests were employed to cover the components to different extent. The first test consisted on the visualization of several images and the posterior writing of the name of the images as they were recalled, focusing on short-term memory. Regarding verbal and non-verbal information, it will be an associative memory because non-verbal information (images) is perceived and must be written in the form of words (verbal) (Novoa et al., 2008).

In this “Image” Test (Test 1), the visuospatial sketchpad is triggered to obtain visual information, and the central executive is utilized to coordinate this information (Baddeley et al., 2011). The storage components are employed by memorizing the images and retrieval is facilitated by annotating them subsequently. Finally, this test also has an associative component, requiring the relation of non-verbal information (images) and subsequent transfer to verbal (words describing the images) (Novoa et al., 2008). This associative component is not utilized in the study by Unsworth et al. (2005) and is a function of the central executive (Baddeley et al., 2011); thus it is of interest to include it.

The second test was based on listening to the elicitation of mathematical operations and their result and determining if the result is correct (“Math” Test) (similar to Unsworth et al., 2005). In their study, they employed the computerized operations interval task to measure working memory. This task involved presenting examinees with a series of simple arithmetic operations, which they must either solve correctly or incorrectly, and decide if they were true or false. Following each operation, individuals were presented with a letter, which they had to remember. Accordingly, the following components were distinguished: processing (arithmetic operation), decision (true or false), storage (letter) and retrieval (array of letters). In contrast, the study by Novoa et al. (2008) also measures associative memory, as it requires the relating of verbal and non-verbal information.

The type of working memory that is measured in this test is verbal because the operations are numbered, and the answers are written with words. This is an immediate memory because they answer immediately after listening to each operation. Moreover, there is a processing component for arithmetic operations and a decision component for choosing between true and false. An auditory component was also present because the operations were listened to.

In order to perform the “Math” Test (Test 2), the phonological loop is triggered, which elicits auditory verbal information, and so is the central executive, which coordinates such information to obtain the true or false response (Baddeley et al., 2011). Furthermore, the processing components are utilized, as they involve arithmetic operations, and decision-making, as they require the selection between true and false.

#### 2.1.1 Test 1: image recovery [T1]

**Figure 1:**
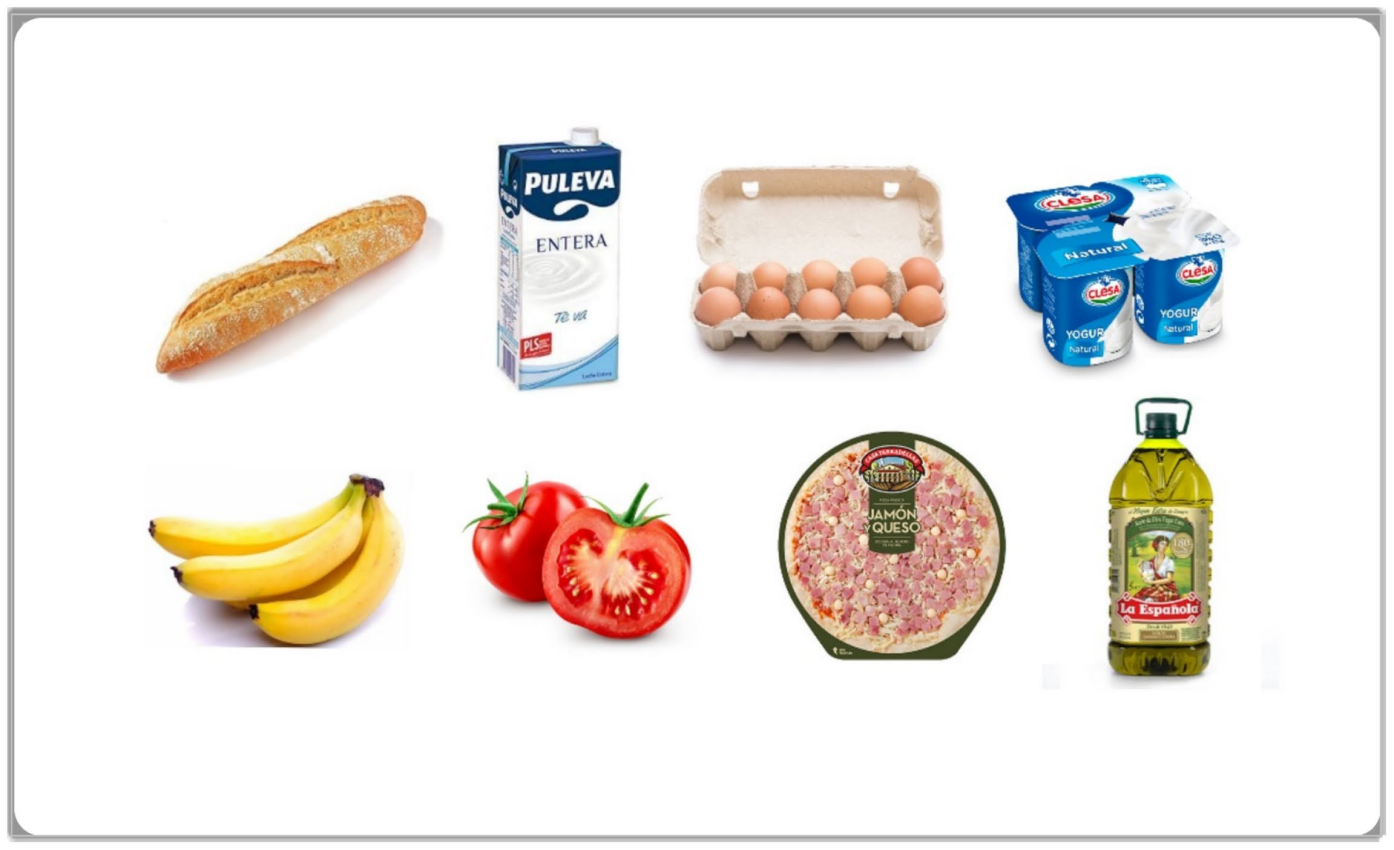
Images of the “Image” test (El Mimbre, 2022; Amazon, 2022; De Diego Ramos, 2018; Clesa, 2022; Foro Química y Sociedad, 2015; Quirónsalud, 2021; Casa Tarradellas, 2022; La Española Aceites, 2022).

The test consisted of two parts. First, one screenshot with 8 images was shown. The number was selected since short-term memory allows to recollect and maintain 7 ± 2 items, namely, between five and nine items (Miller, 1956). Typical food was chosen from the Spanish shopping list because it was related to and known by the participants (see Figure 1).

The exposition lasted 30 s so the participants had time to pay attention to each of the images for 3.5 s. A short duration was selected to avoid the repetition effect, which converts short-term memory into long-term memory (Salkind, 2005).

Second, for the recovery test, the experimental subjects had to write the images they remembered for 30 s. To determine the number of images correctly recovered, their names did not have to be in the same order as they were on the screen. Also, word or word sets that defined each image were admitted as correct answers. Similarly, misspellings were admitted because this dimension was not measured in this experiment.

Accordingly, the score of each participant in Test 1 was a random variable, “ImagesOK” whose range was from 0 to 8, in terms of the number of correct images recovered out of the 8 shown.

- ImagesOK ∊ {0, 1, …, 7, 8}

#### 2.1.2 Test 2: arithmetic operations [T2]

Any participant was required to write if eight simple arithmetic operations were true or false. For this research, and because Test 2 was administered in between the image display and the image recovery, just eight simple arithmetic operations were chosen so the whole test only lasted 1 min (and average of 4 s to listen to the operation and 3 s to answer).

The arithmetic operations and the way they read were as follows:

- 3 + 7 = 9: “Tres más siete es igual a nueve.” (It is false).
- 5–2 = 3: “Cinco menos dos es igual a tres.” (It is true).
- 9 + 4 = 15: “Nueve más cuatro es igual a quince.” (It is false).
- 5–1 = 4: “Cinco menos 1 es igual cuatro.” (It is true).
- 6 + 4 = 10: “Seis más cuatro es igual a diez.” (It is true).
- 9–5 = 3: “Nueve menos cinco es igual a tres.” (It is false).
- 8 + 3 = 11: “Ocho más tres es igual a once.” (It is true).
- 8–6 = 4: “Ocho menos seis es igual a cuatro.” (It’s false).

These operations were read loud, clear, and leisurely. For the TRUE answer “V,” “verdad,” “verdadero,” “sí,” “T,” “true,” and any abbreviation for this word were acceptable. For the false answer, “F,” “Falso,” “false,” “no” and any abbreviation of these words was acceptable. Additionally, misspelled words were allowed.

Accordingly, the score of each participant in Test 2 was a random variable, “MathOK,” whose range was from 0 to 8, in terms of the number of correct true/false answers provide to the 8 operations.

- MathOK ∊ {0, 1, …, 7, 8}

### 2.2 Chronic exercise

To account for chronic exercise, the participants had to answer in words a simple direct question about the time they spent doing exercise per week.

“How many hours per week do you dedicate to exercise?”

For the analysis of the effect of chronic exercise on working memory, the provided amount was a continuous variable that was readily studied. This variable was also categorized, in this case, following the guidelines of the World Health Organization (WHO). WHO recommends that adults between the ages of 18 and 64 engage in moderate aerobic physical tests for at least 2.5 to 5 h per week or intense aerobic physical tests for at least 1.25 to 2.5 h per week [World Health Organization (WHO), 2024].

Four different categorical variables were set for this study, following the continuous declared answer and the WHO thresholds.

- Active ∊ {0 = Sedentary, less than 30 min/week; 1 = Active, more than 30 min/week}
- WHO-2 ∊ {1 = less than 1.25 h/week; 2 = more than 1.25 h/week}
- WHO-3 ∊ {1 = less than 1.25 h/week; 2 = recommended between 1.25 and 5 h/week, 3 = more than 5 h/week}
- WHO-4 ∊ {1 = less than 1.25 h/week; 2 = recommended between 1.25 and 5 h/week, 3 = more than 5 h/week, 4 = more than 10 h/week}

The first variable, Active, differentiates between those that are sedentary and those who engage in chronic exercise. The former group, comprising those who do not perform physical exercise for at least 30 min per week, can be distinguished from the latter group, which includes those who do perform physical exercise for at least 30 min per week.

The second variable with 2 categories, WHO-2, discriminates between those individuals who do not meet the recommended weekly physical test guidelines, defined as less than 1.25 h per week, and those that do fulfil the minimum threshold.

The third variable with three categories, WHO-3, is the one that directly relates to both WHO lower and upper thresholds. The variable separates individuals who exactly meet the recommended weekly physical test guidelines within the range of 1.25 to 5 h, from those that under-exercise and also from those that over-exercise.

Finally, the fourth variable with 4 categories, WHO-4, uses the lower two categories of WHO-3 and splits the one that over-exercises into two, separating those who perform up to twice the maximum recommended weekly physical test, 5 to 10 h, from those that exercise even more than 10 h.

### 2.3 Acute exercise: high-intensity functional training (HIFT)

To assess the influence of acute physical exercise, the subgroup of participants performed intense physical exercise immediately before any measurement test.

In this research, High-Intensity Functional Training HIFT was selected [like Wilke (2020)]. There are studies on the potential relationship between functional fitness training and working memory (the rationale for this study). Nevertheless, physical exercise, encompassing both aerobic and strength training, has been demonstrated to enhance a range of cognitive functions, including executive functions, particularly working memory (Ben-Zeev and Okun, 2021).

HIFT is a type of functional training (that is, a focus on both strength and resistance exercises). HIFT incorporates both aerobic and strength exercises performed at high intensity. The objective of this training is to enhance cardiovascular endurance, strength, body composition, agility, speed, power, and strength (Buckley et al., 2015; Feito et al., 2018). This training modality was created in 1995 in the United States by Greg Glassman and was designed to develop strength, cardiovascular or respiratory endurance, flexibility, coordination, speed, agility, balance, and precision. The functional movements performed were varied and at a high intensity, utilizing advanced gymnastic exercises, weightlifting, and cardiovascular activities (Hernández et al., 2022).

The physical exercise for this research was designed by a HIFT expert to last 1 h, that is, the length of a regular training session at any gym. First, the participants warmed up, before repeating the following 6 exercises three times in the same sequence.

1. 10 reps deadlift.
2. 10 reps power clean and jerk.
3. 10 reps kettlebells swing.
4. 10 reps dumbbell snatch with the left hand.
5. 10 reps dumbbell snatch with the right hand.
6. 10 reps burpee.

Then, they completed 3 training windows of 5 min each. These windows were done twice in the same sequence. When they finished the three windows, they repeated them again.

Window 1:

1. 15 reps deadlift.
2. Maximum reps burpee.
3. From 3 to 6 reps power clean and jerk.
4. Rest.
5. Maximum reps sit ups.

Window 2:

1. 200 meters running.
2. Maximum reps dumbbell SNATCH with the right hand.
3. Maximum reps dumbbell snatch with the left hand.
4. Maximum reps dumbbell snatch with both hands.
5. Maximum reps sit ups.

Window 3:

1. 30 reps double under rope (every time the person jumps, the rope must pass under their feet twice) + maximum reps Burpees.
2. 10 reps bar muscle ups.
3. Maximum reps kettlebells swing.
4. Maximum time doing Grip Hold (the person must hang from the bar with their hands, having arms and legs stretched out).
5. Maximum reps sit ups.

For the analysis of the effect of intense acute physical exercise on working memory, a control group was implemented with participants that had not performed any physical activity in the previous two hours.

Therefore, one categorical variable, “Acute,” was set for the analysis, with two distinct categories:

- Acute ∊ {0 = No; 1 = Yes}

### 2.4 Experiment

With the appraisal tests available and the variables that categorize the physical activity, acute or chronic exercise, also in place, what follows is the explanation of how the experiment was carried out and analyzed, following the protocols approved by the Ethics in Research Committee of the Universidad Rey Juan Carlos.

The experimental group was randomly selected among subjects taking 1-h training sessions at the gym where the instructor was the trainer that designed the HIFT exercise included in this research. Five sessions were consecutively held from 5 pm to 10 pm in the same day. The subjects were asked to participate in the study right after each HIFT session.

Similarly, subjects were randomly selected at the entrance of the gym to participate in the study as the control group, with the stipulation that they had not performed any physical exercise for a period of two hours preceding the experiment.

In both cases, the subjects had to be of legal age and of working age in Spain, that is, between 16 and 65 years old. Moreover, the subjects were required to be free of any medical conditions that would prevent them from engaging in physical activity; that is to say, they were required to be in good health.

#### 2.4.1 Execution

To get matters started, the participants received a sheet of paper and answered first the question related to how often they regularly exercise, quantified in hours per week. Afterwards, the measurement of working memory started with the visualization of 8 images on a projector/laptop screen for 30 s, which is the first part of Test 1: “Image Recovery”.

Subsequently, individuals performed Test 2: “Math test.” Participants listened to each mathematical sentence for about 4 s and had 3 s to write the true/false answer in the same sheet of paper as the previous test.

Just after three seconds of the last answer, individuals finished Test 1 (“Image Recovery”). For this test, the subjects had to write the images they remembered. They had to write their answers on the same sheet of paper next to the answers of the previous test. After 30 s, the sheets were removed and stored.

#### 2.4.2 Data analysis

For testing the statistical significance of the different hypothesis of the research, that is, to corroborate that physical activity (acute or chronic exercise) favors working memory, traditional one-way ANOVA analysis was carried out. For each of the two appraisal tests (image recovery and math) that measure working memory, the *p*-values for significance and the pη^2^ indicator for effect size were calculated [as per Deodato et al. (2024)]. Significance was set at 0.05 following APA standards. Two-way ANOVA analysis was also carried out to try to understand which type of exercise better explained the results of the test.

## 3 Results

### 3.1 Overall

The total sample consisted of 73 volunteer participants over 18 years of age. The experimental group, i.e., the group of participants who underwent functional fitness training immediately prior to the working memory measurement consisted of 29 participants who regularly received classes in the gym where the experiment was conducted. On the other hand, the control group, consisting of the participants who did not perform the functional fitness training, consisted of 44 participants. It is noteworthy that the sample size aligns with the 36 articles incorporated into the extant meta-analysis by Gilson et al. (2023). The sample size in the meta-analysis ranges from 11 to 91 (average of 38.54), with the exception of one study comprising 945 participants, obtained from the “Generation 100 Study” (Zotcheva et al., 2022). Moreover, 75% of the articles report a sample size smaller than the 73 of this research.

#### 3.1.1 Working memory tests

Regarding the “Image” Test (Test 1), the mean number of correct responses was 6.93, with a maximum of 8 correct responses obtained by 27 individuals (36.99% of the sample), and a minimum of 4 correct responses obtained by 2 subjects (2.74% of the sample) (see Table 1).

**Table 1:** Summary of correct answers to the tests.

Correct answers	Image recovery test		Mathematical test	
	Participants	Percentage	Participants	Percentage
8	27	36.99%	60	82.19%
7	24	32.88%	11	15.07%
6	14	19.18%	1	1.37%
5	6	8.22%		
4	2	2.74%		
3			1	1.37%
TOTAL	73	100.00%	73	100.00%

About the “Math” Test (Test 2), the mean number of correct responses was 7.75, with a maximum of 8 correct responses obtained by 60 individuals (82.19% of the sample), and a minimum of 3 correct responses obtained by one subject (1.37% of the sample).

#### 3.1.2 Chronic exercise

The weekly hours dedicated by each participant per week were extracted from the questionnaire, ranging from 0 to 16 h, with an average of 5.98 h per week. The following table shows the frequency of each number of weekly hours of exercise per week (see Table 2).

**Table 2:** Summary of chronic exercise in hours per week based on the World Health Organization (WHO) classification.

Class (hours/week)	Chronic			
	Active	WHO-2	WHO-3	WHO-4
None (0)	9	11	11	11
Below (0–1.25)	64			
Recommended (1.25–5)		62	28	28
Over (5–10)			34	25
Twice Over (10+)				9

Nine participants reported no physical exercise at all, while two others indicated values under the WHO threshold of 1.25. Twenty-eight participants reported values within the recommended range of WHO, leaving thirty-four that indicated over-exercise (twenty-five between 5 and 10 h per week and nine over twice the threshold).

### 3.2 Physical activity and working memory

**Figure 2:**
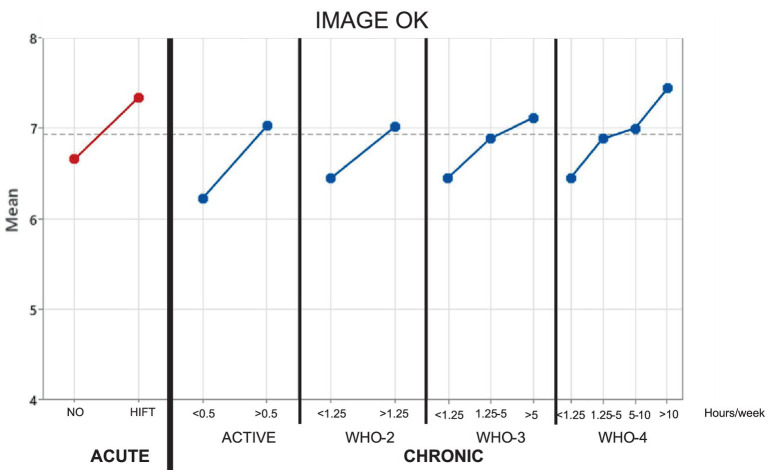
Graphical display of effects on image recovery.

The one-way ANOVA effects on “Image Recovery” of acute exercise (red graph on the left of the main effects plot shown in Figure 2) showed significance (see Table 3, *p*-value = 0.007, pη^2^ = 0.099) whereas that of chronic exercise (four blue graphs in Figure 2) did not for WHO classifications (*p*-value > 0.11). Only that of Active (*p*-value = 0.033, pη^2^ = 0.062) was significant related to chronic exercise. Concerning two-way ANOVA, acute exercise always showed significance whereas chronic exercise did not under any model. No interactions were significant in any case.

**Table 3:** Significance of effects of image recovery (*p-value <0.05).

*p*-value; pη^2^	Acute	Chronic			
		Active	WHO-2	WHO-3	WHO-4
One-way	*p*-value = 0.007*; pη^2^ = 0.099	*p*-value = 0.033*; pη^2^ = 0.062	*p*-value = 0.110; pη^2^ = 0.036	*p*-value = 0.199; pη^2^ = 0.045	*p*-value = 0.225; pη^2^ = 0.061
Two-way	*p*-value = 0.028*; pη^2^ = 0.065	*p*-value = 0.153; pη^2^ = 0.027			
	*p*-value = 0.017*; pη^2^ = 0.078		*p*-value = 0.334; pη^2^ = 0.012		
	*p*-value = 0.016*; pη^2^ = 0.079			*p*-value = 0.416; pη^2^ = 0.023	
	*p*-value = 0.021*; pη^2^ = 0.073				*p*-value = 0.474; pη^2^ = 0.033

The one-way ANOVA effects on “Image Recovery” of acute exercise (red graph on the left of the main effects plot shown in Figure 2) showed significance (see Table 3, *p*-value = 0.007, pη^2^ = 0.099) whereas that of chronic exercise (four blue graphs in Figure 2) did not for WHO classifications (*p*-value > 0.11). Only that of Active (*p*-value = 0.033, pη^2^ = 0.062) was significant related to chronic exercise. Concerning two-way ANOVA, acute exercise always showed significance whereas chronic exercise did not under any model. No interactions were significant in any case.

**Figure 3:**
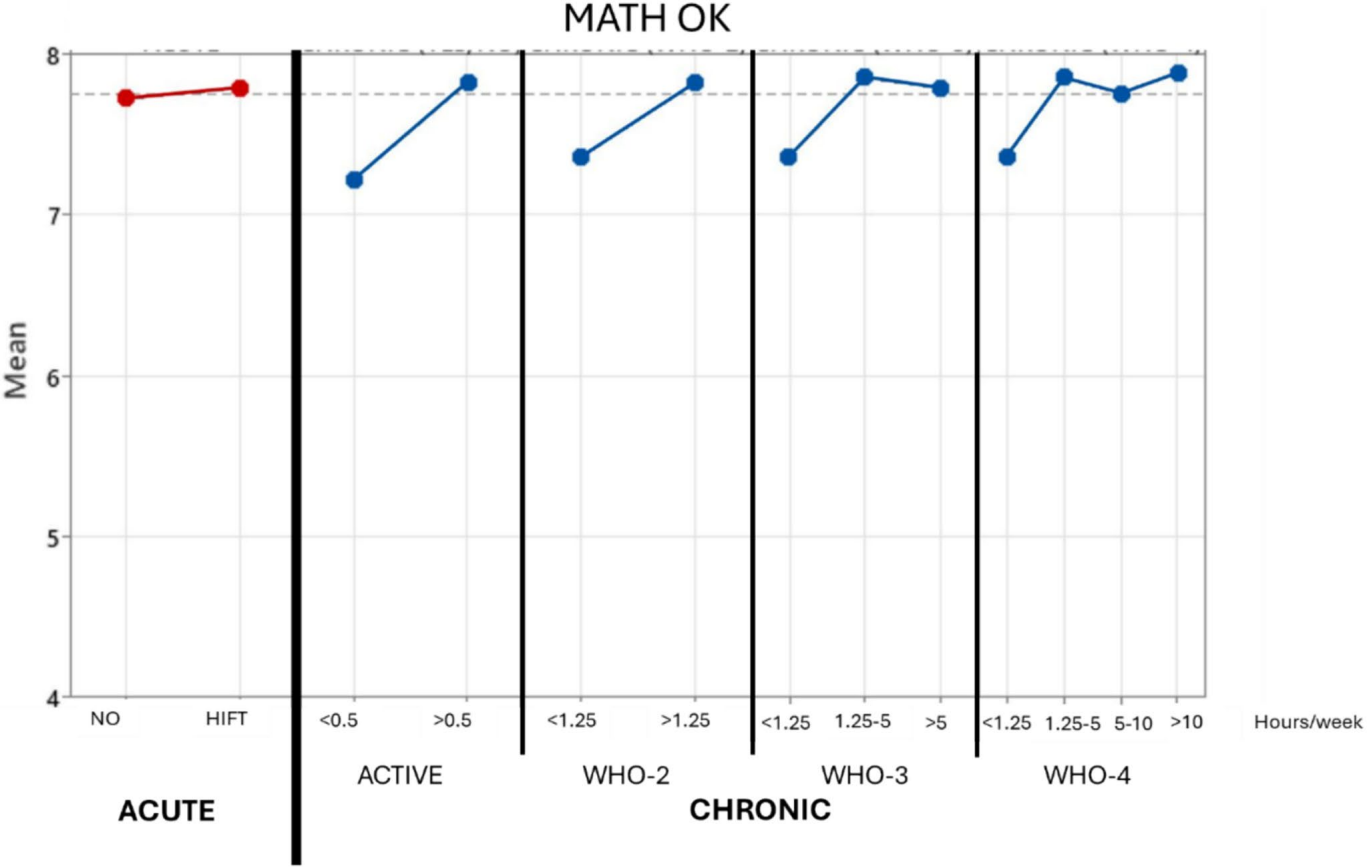
Graphical display of effects on math processing.

Regarding “Math,” although the average scores were lower in the case of those that did not carry the HIFT training out, there was no significant effect of acute exercise (see Table 4, *p*-value = 0.698; pη^2^ = 0.002). There was also no significant effect of chronic exercise on mathematical processing for WHO-3 and for WHO-4 but there was a significant effect if only 2 categories were used (WHO-2, *p*-value = 0.045; pη^2^ = 0.055), differentiating those that do not reach the recommended WHO minimum value of 1.25 h per week and those that perform more. The significant effect in this last model was also present when the two-way ANOVA was performed. No interactions were significant in any case (see Figure 3).

**Table 4:** Significance of effects on math processing.

*p*-value; pη^2^	Acute	Chronic			
		Active	WHO-2	WHO-3	WHO-4
One-way	*p*-value = 0.698; pη^2^ = 0.002	*p*-value = 0.014*; pη^2^ = 0.081	*p*-value = 0.045*; pη^2^ = 0.055	*p*-value = 0.128; pη^2^ = 0.057	*p*-value = 0.230; pη^2^ = 0.06
Two-way	*p*-value = 0.710; pη^2^ = 0.002	*p*-value = 0.015*; pη^2^ = 0.081			
	*p*-value = 0.888; pη^2^ = 0.000		*p*-value = 0.050*; pη^2^ = 0.054		
	*p*-value = 0.882; pη^2^ = 0.000			*p*-value = 0.140; pη^2^ = 0.055	
	*p*-value = 0.845; pη^2^ = 0.001				*p*-value = 0.247; pη^2^ = 0.059

## 4 Discussion

**Figure 4:**
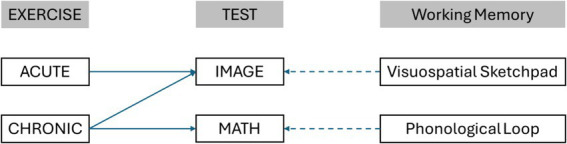
The effects of exercise on the components of working memory.

Figure 4 summarizes the findings related to the effects of acute and chronic exercise on the components of working memory, highlighting the importance of performing acute exercise before carrying a task with a high impact on the visuospatial sketchpad and not on the phonological loop whereas chronic exercise affects both components of the working memory.

In the case of ACUTE exercise [H1], it can be confirmed that the previous performance of a 1-h acute functional fitness training improved the component of the working memory measured through the “Image” Test (Test 1: the recall of previously exposed images with the visuospatial sketchpad), but not that measured through the “Math” Test (Test 2: the resolution of simple arithmetic operations mainly with the phonological loop). This is in accordance with the findings of the previous studies, which concluded that short sessions of physical exercise improve cognition (Wilke, 2020; Hsieh et al., 2020; Ludyga et al., 2016).

The results also indicate that CHRONIC exercise improves working memory [H2]. This improvement was significant for the recovery of previously exposed images (Test 1) as well as for the performance of simple arithmetic operations (Test 2). By dividing the sample according to the objectives set by WHO, we can confirm that this relationship also exists. In the case of arithmetic operations (Test 2), the fulfilment or non-fulfilment of these objectives is more important than in the case of image recovery (Test 1). However, in both tests, it could be said that there is a positive effect of chronic exercise on working memory. Our results were also aligned with Hsieh et al. (2020), who found that chronic exercise significantly improves working memory, and Li et al. (2024), who concluded the same with adolescences. Other studies, however, demonstrated either small improvements or non-significant results (Herold et al., 2022).

It is therefore worth highlighting that the main advancement in this research is that the effects of different types of exercise have been separated and fully analyzed for the first time to our knowledge between two of the components of the working memory, with acute exercise performed immediately before the cognitive tasks impacting only on the visuospatial sketchpad, whereas chronic exercise impacts both on the visuospatial sketchpad and on the phonological loop. In other words, the conclusion is that different types of exercise affect different aspects of the working memory.

Regarding the implications, we can state that it would be beneficial for health in general and cognition in particular to perform chronic exercise of different intensities. In fact, schools and universities should foster exercise of any sort. Besides, acute exercise should be performed just before performing a visual task, so working centers could set rooms to facilitate the practice of intense exercise (not necessarily HIFT).

As a summary, this study stands out for its use of a specifically designed one-hour acute HIFT training protocol, an approach that has been little explored in the literature on exercise and cognition. Another significant difference lies in the duration of this acute exercise, which was set at 60 min, whereas the studies included in the previous meta-analysis were carried out in sessions that varied between 6 and 40 min (Mou et al., 2022; Wilke, 2020; Solianik et al., 2021; Nasrollahi et al., 2022; Xie et al., 2020), which makes it possible to evaluate the effects of a longer intervention on working memory, with the duration that a workout usually takes in gyms today.

As for the tests used to measure working memory, the study includes an arithmetic task similar to that used by Unsworth et al. (2005), as well as a novel test based on *ad hoc* selected images. The latter represents a methodological innovation, similar to other tasks used in previous studies that evaluate the memory of working with letters or words, but with the advantage of being inclusive of people who speak different languages. Similarly, its design allows for a possible adaptation to assess working memory in children who have not yet acquired reading skills, through oral rather than written responses.

This study also differs in that it compares the effects of recent physical exercise with a state of inactivity, understood as the performance of daily activities without including physical exercise, unlike most previous studies in which the control group performs another type of physical activity (Zhu et al., 2021), such as low-intensity exercise, or even cognitive activities such as reading (Wilke et al., 2019).

Furthermore, the study is based on a large sample size compared to most previous studies, which strengthens the validity of the findings, although it would be beneficial to further expand the sample to improve the generalizability of the results.

Concerning the limitations, the present study did not induce or assess participants’ expectations, so it is possible that the placebo effect may have contributed to the observed results. This is due to the cultural association between physical exercise and cognitive benefits (Chhabra and Szabo, 2024).

Also, the number of hours of physical exercise performed by participants per week was self-reported; therefore, this information may be inaccurate and could be improved in future studies. Indeed, research has identified a modest correlation between self-reported and objectively measured physical activity levels (Prince et al., 2008). A noteworthy finding is that individuals frequently overestimate the frequency of exercise while often underestimating its duration (Matt et al., 1999). These findings address the necessity for more precise and reliable measures of physical activity to evaluate interventions and elucidate relationships between activity and health outcomes.

Besides, extant studies suggest that as the duration of exercise increases, the cognitive benefits may be counterbalanced by the effects of fatigue (Boere et al., 2025). In fact, Hsieh et al. (2020) stated that a brief period of physical test was more efficient than a longer one in enhancing cognitive performance. It would be of interest to vary the duration of the training session to ascertain whether the improvements observed are even more pronounced. This would permit an analysis of the optimal length of functional fitness training for the improvement of working memory.

Finally, and even if we have followed previous research in the design of the appraisal tests, we are aware that they might be modified regarding the number of images and their color and size in Test 1 and the number of math equations and their difficulty in Test 2.

For future research, then, besides increasing the sample, it would also be interesting to vary the length of the training as well as the difficulty of the appraisal tests. On that regard, other statistical analysis related to the appraisal of treatments could be used, for example, dual-task procedures (Deodato et al., 2024). It is also recommended that more robust measures of physical activity be employed, such as accelerometers, heart rate monitors, or oximeters.

In conclusion, this research based on cognitive psychology clearly indicates that physical activity, both through acute and chronic exercise, helps improve mental health and cognitive flexibility measured by working memory and its components.

## Data Availability

The datasets presented in this article are not readily available because they could compromise the privacy of research participants according to the Ethical Committee of the URJC. Requests to access the datasets should be directed to the corresponding author Eva Borrega-Alonso: eva.borrega@urjc.es.

## References

[ref9001] Amazon. (2022). Puleva Leche Entera Brick [Image]. Available at: https://www.amazon.es/Puleva-Leche-Entera-Brick/dp/B019Y4II92 (Accessed May 20, 2025).

[ref1] APA Dictionary of Psychology. (2025) APA Dictionary of Psychology. Available online at: https://dictionary.apa.org/ [Accessed April 1, 2025].

[ref3] Baddeley A. (2012). Working memory: theories, models, and controversies. Annu. Rev. Psychol. 63, 1–29. doi: 10.1146/annurev-psych-120710-100422, PMID: 21961947

[ref2] Baddeley A. D. (2010). Working memory. Curr. Biol. 20, R136–R140. doi: 10.1016/j.cub.2009.12.014, PMID: 20178752

[ref4] Baddeley A. D. Allen R. J. Hitch G. J. (2011). Binding in visual working memory: the role of the episodic buffer. Neuropsychologia 49, 1393–1400. doi: 10.1016/j.neuropsychologia.2010.12.042, PMID: 21256143

[ref5] Ben-Zeev T. Okun E. (2021). High-intensity functional training: molecular mechanisms and benefits. NeuroMolecular Med. 23, 335–338. doi: 10.1007/s12017-020-08638-8, PMID: 33386577

[ref6] Boere K. Copithorne F. Krigolson O. E. (2025). The impact of a two-hour endurance run on brain activity monitored over 24 h. Exp. Brain Res. 243:101. doi: 10.1007/s00221-025-07056-1, PMID: 40126627

[ref7] Buckley S. Knapp K. Lackie A. Lewry C. Horvey K. Benko C.. (2015). Multimodal high-intensity interval training increases muscle function and metabolic performance in females. Appl. Physiol. Nutr. Metab. 40, 1157–1162. doi: 10.1139/apnm-2015-0238, PMID: 26513008

[ref9003] Casa Tarradellas. (2022). Pizza jamón y queso [Image]. Available at: https://casatarradellas.es/products/pizza-jamon-y-queso/ (Accessed May 20, 2025).

[ref8] Chang Y. Labban J. Gapin J. Etnier J. (2012). The effects of acute exercise on cognitive performance: a meta-analysis. Brain Res. 1453, 87–101. doi: 10.1016/j.brainres.2012.02.068, PMID: 22480735

[ref9] Chhabra B. Szabo A. (2024). Placebo and nocebo effects on sports and exercise performance: a systematic literature review update. Nutrients 16:1975. doi: 10.3390/nu16131975, PMID: 38999724 PMC11243088

[ref9002] Clesa. (2022). Yogur natural 4x125g [Image]. Available at: https://soria.e-leclerc.es/detalle/-/Producto/yogur-natural-4x125g/0000084114174 (Accessed May 20, 2025).

[ref10] Connelly L. J. Bailey S. J. Krustrup P. Fulford J. Smietanka C. Jones A. M. (2017). Effects of self-paced interval and continuous training on health markers in women. Eur. J. Appl. Physiol. 117, 2281–2293. doi: 10.1007/s00421-017-3715-9, PMID: 28932907 PMC5640747

[ref11] Costa K. G. Cabral D. A. Hohl R. Fontes E. B. (2019). Rewiring the addicted brain through a psychobiological model of physical exercise. Front. Psych. 10:600. doi: 10.3389/fpsyt.2019.00600, PMID: 31507468 PMC6718472

[ref12] De Asteasu M. L. S. Martínez-Velilla N. Zambom-Ferraresi F. Casas-Herrero Á. Izquierdo M. (2017). Role of physical exercise on cognitive function in healthy older adults: a systematic review of randomized clinical trials. Ageing Res. Rev. 37, 117–134. doi: 10.1016/j.arr.2017.05.007, PMID: 28587957

[ref9004] De Diego Ramos G. (2018). ¿Qué huevos comprar? Estos son los mejores del súper [Online article with image]. Alimente - El Confidencial. Available at: https://www.alimente.elconfidencial.com/consumo/2018-03-21/comprar-mejores-huevos-supermercado_1480507/ (Accessed May 20, 2025).

[ref13] De Greeff J. W. Bosker R. J. Oosterlaan J. Visscher C. Hartman E. (2018). Effects of physical test on executive functions, attention and academic performance in preadolescent children: a meta-analysis. J. Sci. Med. Sport 21, 501–507. doi: 10.1016/j.jsams.2017.09.595, PMID: 29054748

[ref14] Deodato M. Granato A. Buoite Stella A. Martini M. Marchetti E. Lise I.. (2024). Efficacy of a dual task protocol on neurophysiological and clinical outcomes in migraine: a randomized control trial. Neurol. Sci. 45, 4015–4026. doi: 10.1007/s10072-024-07611-8, PMID: 38806882 PMC11255006

[ref15] Diamond A. (2013). Executive functions. Annu. Rev. Psychol. 64, 135–168. doi: 10.1146/annurev-psych-113011-143750, PMID: 23020641 PMC4084861

[ref16] Eather N. Riley N. Miller A. Smith V. Poole A. Vincze L.. (2019). Efficacy and feasibility of HIIT training for university students: the Uni-HIIT RCT. J. Sci. Med. Sport 22, 596–601. doi: 10.1016/j.jsams.2018.11.016, PMID: 30509862

[ref9006] El Mimbre. (2022). Gallega barra 240 g [Image]. Available at: https://elmimbreonline.com/productos/gallega-barra-240-g (Accessed May 20, 2025).

[ref17] Feito Y. Heinrich K. Butcher S. Poston W. (2018). High-intensity functional training (HIFT): definition and research implications for improved fitness. Sports 6:76. doi: 10.3390/sports6030076, PMID: 30087252 PMC6162410

[ref9008] Foro Química y Sociedad. (2015). Plátanos para ser feliz (y mucho más) [Image]. Available at: https://www.quimicaysociedad.org/platanos-para-ser-feliz-y-mucho-mas/ (Accessed May 20, 2025).

[ref18] Gilson N. D. Andersson D. Papinczak Z. E. Rutherford Z. John J. Coombes J. S.. (2023). High intensity and sprint interval training, and work-related cognitive function in adults: a systematic review. Scand. J. Med. Sci. Sports 33, 814–833. doi: 10.1111/sms.14349, PMID: 36916717

[ref19] Guiney H. Machado L. (2012). Benefits of chronic aerobic exercise for executive functioning in healthy populations. Psychon. Bull. Rev. 20, 73–86. doi: 10.3758/s13423-012-0345-4, PMID: 23229442

[ref20] Hernández N. Cervantes N. Carrasco C. E. (2022). Comparación de pruebas para medir la fatiga muscular en el entrenamiento de atletas hombres de CrossFit: una revisión sistemática. Retos: nuevas tendencias en educación física, deporte y recreación 43, 923–930. doi: 10.47197/retos.v43i0.89787

[ref21] Herold F. Behrendt T. Meißner C. Müller N. G. Schega L. (2022). The influence of acute Sprint interval training on cognitive performance of healthy younger adults. Int. J. Environ. Res. Public Health 19:613. doi: 10.3390/ijerph19010613, PMID: 35010873 PMC8745010

[ref22] Hillman C. H. Erickson K. I. Kramer A. F. (2008). Be smart, exercise your heart: exercise effects on brain and cognition. Nat. Rev. Neurosci. 9, 58–65. doi: 10.1038/nrn2298, PMID: 18094706

[ref23] Hsieh S. Chueh T. Huang C. Kao S. Hillman C. H. Chang Y.. (2020). Systematic review of the acute and chronic effects of high-intensity interval training on executive function across the lifespan. J. Sports Sci. 39, 10–22. doi: 10.1080/02640414.2020.1803630, PMID: 32780634

[ref24] Kamijo K. Hayashi Y. Sakai T. Yahiro T. Tanaka K. Nishihira Y. (2009). 'Acute effects of aerobic exercise on cognitive function in older Adults', the journals of gerontology. Series B Psychol. Sci. Soc. Sci. 64B, 356–363. doi: 10.1093/geronb/gbp030, PMID: 19363089

[ref25] Kao S. Cadenas-Sanchez C. Shigeta T. T. Walk A. M. Chang Y. Pontifex M. B.. (2019). A systematic review of physical test and cardiorespiratory fitness on P3b. Psychophysiology 57:e13425. doi: 10.1111/psyp.13425, PMID: 31228362

[ref26] Kovacevic A. Fenesi B. Paolucci E. Heisz J. J. (2019). The effects of aerobic exercise intensity on memory in older adults. Appl. Physiol. Nutr. Metab. 45, 591–600. doi: 10.1139/apnm-2019-0495, PMID: 31665610

[ref9009] La Española Aceites. (2022). Aceite de oliva virgen extra Selección Mediterránea 5L [Image]. Available at: https://enda.laespanolaaceites.com/products/aceite-de-oliva-virgen-extra-la-espanola-seleccion-mediterranea-5l (Accessed May 20, 2025).

[ref27] Li J. W. O’Connor H. O’Dwyer N. Orr R. (2017). The effect of acute and chronic exercise on cognitive function and academic performance in adolescents: a systematic review. J. Sci. Med. Sport 20, 841–848. doi: 10.1016/j.jsams.2016.11.025, PMID: 28185806

[ref28] Li Y. Wang F. Li J. Huo X. Zhang Y. (2024). Aerobic exercise improves verbal working memory sub-processes in adolescents: behavioral evidence from an N-back task. PeerJ 12:e17331. doi: 10.7717/peerj.17331, PMID: 38708349 PMC11067889

[ref29] Ludyga S. Gerber M. Brand S. Holsboer-Trachsler E. Pühse U. (2016). Acute effects of moderate aerobic exercise on specific aspects of executive function in different age and fitness groups: a meta-analysis. Psychophysiology 53, 1611–1626. doi: 10.1111/psyp.12736, PMID: 27556572

[ref30] Mandolesi L. Polverino A. Montuori S. Foti F. Ferraioli G. Sorrentino P.. (2018). Effects of physical exercise on cognitive functioning and wellbeing: biological and psychological benefits. Front. Psychol. 9:509. doi: 10.3389/fpsyg.2018.00509, PMID: 29755380 PMC5934999

[ref31] Matt G. E. Garcia M. Primicias W. W. Frericks L. de Fabia F. (1999). Exploring biases in self-reported exercising behavior: heuristics based on Recency, frequency, and preference. Percept. Mot. Skills 88, 126–128. doi: 10.2466/pms.1999.88.1.126, PMID: 10214638

[ref32] McMorris T. Sproule J. Turner A. Hale B. J. (2011). Acute, intermediate intensity exercise, and speed and accuracy in working memory tasks: a meta-analytical comparison of effects. Physiol. Behav. 102, 421–428. doi: 10.1016/j.physbeh.2010.12.007, PMID: 21163278

[ref33] Miller G. A. (1956). The magical number seven, plus or minus two: some limits on our capacity for processing information. Psychol. Rev. 63, 81–97. doi: 10.1037/h0043158, PMID: 13310704

[ref35] Mou H. Tian S. Fang Q. Qiu F. (2022). The immediate and sustained effects of moderate-intensity continuous exercise and high-intensity interval exercise on working memory. Front. Psychol. 13:766679. doi: 10.3389/fpsyg.2022.766679, PMID: 35242075 PMC8887601

[ref36] Nasrollahi N. Quensell J. Machado L. (2022). Effects of a brief stair-climbing intervention on cognitive functioning and mood states in older adults. J. Aging Phys. Act. 30, 455–465. doi: 10.1123/japa.2021-0125, PMID: 34510025

[ref37] Novoa A. M. Juárez O. Nebot M. (2008) 'Efectividad de las intervenciones cognitivas en la prevención del deterioro de la memoria en las personas mayores sanas', Rev. Esp. Salud Publica. Available online at: https://scielo.isciii.es/scielo.php?script=sci_arttext&pid=S0213-91112008000500013 10.1157/13126930 19000530

[ref38] Prince S. A. Adamo K. B. Hamel M. Hardt J. Connor Gorber S. Tremblay M. (2008). A comparison of direct versus self-report measures for assessing physical activity in adults: a systematic review. Int. J. Behav. Nutr. Phys. Act. 5:56. doi: 10.1186/1479-5868-5-56, PMID: 18990237 PMC2588639

[ref39] Quintero A. P. Bonilla-Vargas K. J. Correa-Bautista J. E. Domínguez-Sanchéz M. A. Triana-Reina H. R. Velasco-Orjuela G. P.. (2018). Acute effect of three different exercise training modalities on executive function in overweight inactive men: a secondary analysis of the BrainFit study. Physiol. Behav. 197, 22–28. doi: 10.1016/j.physbeh.2018.09.010, PMID: 30248301

[ref9005] Quirónsalud. (2021). The tomato and its health benefits [Image]. Available at: https://www.quironsalud.com/san-jose/es/sala-prensa/actualidad/tomate (Accessed May 20, 2025).

[ref40] Rathore A. Lom B. (2017). The effects of chronic and acute physical test on working memory performance in healthy participants: a systematic review with meta-analysis of randomized controlled trials. Syst. Rev. 6:124. doi: 10.1186/s13643-017-0514-7, PMID: 28666470 PMC5493123

[ref41] Salkind N. J. (2005). “Long-term memory,” in Encyclopedia of Human Development. ed. Salkind N. J. (Thousand Oaks, CA: Sage). doi: 10.4135/9781412952484.n385

[ref42] Serra L. Raimondi S. Di Domenico C. Maffei S. Lardone A. Liparoti M.. (2021). The beneficial effects of physical exercise on visuospatial working memory in preadolescent children. AIMS Neurosci. 8, 496–509. doi: 10.3934/neuroscience.2021026, PMID: 34877401 PMC8611191

[ref43] Soga K. Masaki H. Gerber M. Ludyga S. (2018). Acute and long-term effects of resistance training on executive function. J. Cogn. Enhanc. 2, 200–207. doi: 10.1007/s41465-018-0079-y

[ref44] Solianik R. Bružas V. Mockus P. Vadopalas K. Streckis V. (2021). Acute effects of high-intensity interval training on cognition and retinal microcirculation in experienced amateur boxers. J. Sports Med. Phys. Fitness 61, 867–873. doi: 10.23736/S0022-4707.20.11352-5, PMID: 33269877

[ref46] Unsworth N. Heitz R. P. Schrock J. C. Engle R. W. (2005). An automated version of the operation span task. Behav. Res. Methods 37, 498–505. doi: 10.3758/bf03192720, PMID: 16405146

[ref48] Wilke J. (2020). Functional high-intensity exercise is more effective in acutely increasing working memory than aerobic walking: an exploratory randomized, controlled trial. Sci. Rep. 10:12335. doi: 10.1038/s41598-020-69139-z, PMID: 32703974 PMC7378555

[ref49] Wilke J. Giesche F. Klier K. Vogt L. Herrmann E. Banzer W. (2019). Acute effects of resistance exercise on cognitive function in healthy adults: a systematic review with multilevel Meta-analysis. Sports Med. 49, 905–916. doi: 10.1007/s40279-019-01085-x, PMID: 30838520

[ref50] World Health Organization (WHO). (2024) 'Actividad física', World Health Organization. Available online at: https://www.who.int/es/news-room/fact-sheets/detail/physical-test

[ref51] Wu J. Qiu P. Li Y. (2024). The association between ‘weekend warrior’, regular exercise, and cognitive function in elderly individuals with and without depressive symptoms: a cross-sectional analysis from NHANES 2011–2014. J. Affect. Disord. 367, 1–7. doi: 10.1016/j.jad.2024.08.184, PMID: 39222850

[ref52] Xie C. Alderman B. L. Meng F. Ai J. Chang Y. K. Li A. (2020). Acute high-intensity interval exercise improves inhibitory control among young adult males with obesity. Front. Psychol. 11:1291. doi: 10.3389/fpsyg.2020.01291, PMID: 32670154 PMC7330122

[ref53] Yuan X. Li D. Hu Y. Qi M. Kong Y. Zhao C.. (2023). Neural and behavioral evidence supporting the relationship between habitual exercise and working memory precision in healthy young adults. Front. Neurosci. 17:1146465. doi: 10.3389/fnins.2023.1146465, PMID: 37090810 PMC10116001

[ref54] Zhang Y. Zhang B. Gan L. Ke L. Fu Y. di Q.. (2021). Effects of online bodyweight high-intensity interval training intervention and health education on the mental health and cognition of sedentary young females. Int. J. Environ. Res. Public Health 18:302. doi: 10.3390/ijerph18010302, PMID: 33401605 PMC7795383

[ref55] Zhu Y. Sun F. Chiu M. M. Siu A. Y. (2021). Effects of high intensity interval exercise and moderate intensity interval exercise on executive function of healthy young males. Physiol. Behav. 239:113505. doi: 10.1016/j.physbeh.2021.113505, PMID: 34153324

[ref56] Zotcheva E. Haberg A. Wisloff U. Salvesen Ø. Selbæk G. Stensvold D.. (2022). Effects of 5 years aerobic exercise on cognition in older adults: the generation 100 study: a randomised controlled trial. Sports Med. 52, 1689–1699. doi: 10.1007/s40279-021-01608-5, PMID: 34878637 PMC9213353

